# Utilizing RNA nanotechnology to construct negatively charged and ultrasound-responsive nanodroplets for targeted delivery of siRNA

**DOI:** 10.1080/10717544.2022.2026532

**Published:** 2022-01-17

**Authors:** Lu Guo, Dandan Shi, Mengmeng Shang, Xiao Sun, Dong Meng, Xinxin Liu, Xiaoying Zhou, Jie Li

**Affiliations:** Department of Ultrasound, Qilu Hospital of Shandong University, Jinan, People’s Republic of China

**Keywords:** Ultrasound nanodroplet, siRNA delivery, tumor-targeting, gene nanotechnology, negatively charged

## Abstract

Ultrasound nanodroplets (NDs) have been reported as a promising nanocarrier for siRNA delivery depending on its unique strengths of sonoporation. Presently, common means for NDs-mediated siRNA delivery is through electrostatic interaction, but challenges like cationic toxicity still exist. In this study, we demonstrated a novel strategy to construct negatively charged and ultrasound (US)-responsive O-carboxymethyl chitosan (O-CMS) NDs as a siRNA targeted delivery system through three-way junction of bacteriophage phi29 DNA packaging motor (3WJ-pRNA) nanotechnology. 39nt A10-3.2 aptamer targeting prostate specific membrane antigen (PSMA) and 21nt siRNA against cationic amino acid transporter 1 (siCAT-1) were annealed to 3WJ-pRNA scaffold via complementation with an extended sequence. The cholesterol molecule attached to one branch facilitates the 3WJ-pRNA nanoparticles anchoring onto NDs. The desired O-CMS NDs with siRNA-loading and RNA-aptamer modification (A10-3.2/siCAT-1/3WJ-NDs) were successfully prepared, which were with spherical shapes, core–shell structures and uniform in sizes (198 nm with PDI 0.3). As a main proportion of shell, O-CMC showed a certain anti-tumor effects. *In vitro* studies demonstrated that A10-3.2/siCAT-1/3WJ-NDs exhibited good contrast-enhanced US imaging, buffering capacity and high bio-safety, were able to deliver siCAT-1 to PSMA-overexpressed prostate cancer cells under US irradiation, thus silence the CAT-1 expression, and consequently suppressing 22RV1 cell proliferation and migration. Taken overall, our findings provide a promising strategy to develop negatively charged and US-responsive NDs for tumor-targeted siRNA delivery.

## Introduction

siRNA-based gene therapies are expected to be effective therapeutic strategies for tumor disease, due to its advantages such as easy to design and able to target every pathogenic gene (Rossi & Rossi, 2021). However, naked siRNA is likely to bind with serum protein and easy to degrade in body’s blood circulation, which remains the bottleneck for clinical application (Charbe et al., 2020; Yoo et al., 2021). Therefore, a safe, efficient delivery system is crucial for further use of siRNA-based therapies. Ultrasound (US) exposure alone had been proven to increase cell membrane permeability and facilitate the delivery of nucleic acids into cells, and addition of US-responsive nanodroplets (NDs) has a significant potentiating effect, leading to enhanced gene uptake because of the sonoporation mechanism (Shi et al., 2017; Chowdhury et al., 2020). Accordingly, the combination of precise US exposure and NDs might be useful for target site-specific siRNA delivery. Through electrostatic interactions, negatively charged siRNA can readily condense with positively charged NDs, which is a common strategies for NDs-mediated siRNA delivery. Despite the success of loading and delivering siRNA, it is worth noting that cationic toxicity is a barrier for further use of these positively charged nanocarriers (Liu et al., 2018; Tai, 2019; Weiss et al., 2021).

Compared to the positively charged NDs that rely on electrostatic interaction for siRNA loading, modulation of siRNA lipophilicity provides another option for incorporating siRNA into neutrally charged or negatively charged NDs (Haraszti et al., 2018; Tai, 2019). In contrast with positively charged NDs, neutrally charged or negatively charged NDs can avoid cationic toxicity such as interaction with leucocytes or serum protein. Due to the lipophilic nature, cholesterol moiety of chol-siRNA is capable to anchor siRNA onto the membrane outlet of liposome and exosome (Haraszti et al., 2018; Tai, 2019). Given that the structure of NDs is similar to liposome or exosome, it can be assumed that chol-siRNA may also be able to anchor onto the membrane outlet of NDs despite the lack of related research. The modification of ligands on the surface of the NDs is critical for achieving tumor-targeted delivery of siRNA. Recently, RNA nanotechnology such as 3WJ-pRNA has been proven to be a versatile and biocompatible platform for specific targeted therapeutic use (Shu et al., 2011; Guo et al., 2018; Pi et al., 2018; Jasinski et al., 2019; Xu et al., 2019; Ghimire et al., 2020). The three-way junction of the 3WJ-pRNA folds by its intrinsic nature into a planar arrangement with three angles of 60°, 120°, and 180° between helical regions (Pi et al., 2018). The extending arms of 3WJ-pRNA structures could be intelligently replaced with therapeutic module such as siRNAs or miRNAs, targeting modules such as RNA aptamers or folate, and other moieties such as fluorescent probes (Guo et al., 2018).

Herein, we exploited the 3WJ-pRNA structure to construct Cy3-labeled negatively charged and US-responsive A10-3.2/siCAT-1/3WJ-NDs bearing an prostate specific membrane antigen (PSMA)-targeting RNA aptamer and siCAT-1 to silence CAT-1 expression in prostate cancer cells. Compared to common methods such as electrostatic interaction, 3WJ-pRNA scaffold bears remarkable advantages for siRNA loading and delivery, such as ultra-thermostable core, multi-module modification and no cationic toxicity. 39nt A10-3.2 RNA aptamer and 21nt siCAT-1 can be annealed to 3WJ-pRNA scaffold via complementation with an extended sequence. The cholesterol molecule attached to one 3WJ branch facilitates the 3WJ-pRNA nanoparticles anchoring to NDs. *In vitro* studies demonstrated that A10-3.2/siCAT-1/3WJ-NDs were successfully constructed, exhibited good contrast-enhanced ultrasound imaging (CEUI) and high bio-safety, enhanced targeted binding to PSMA-overexpressed 22RV1 cells, thus increased delivery of siCAT-1 for CAT-1 inhibition, and consequently suppressing 22RV1 cells proliferation and migration.

## Materials and methods

### Reagents and materials

O-carboxymethyl chitosan (O-CMC, 100–300 kDa, carboxymethylation degree of 95%, deacetylation degree of 90%) was supplied by Santa Cruz Biotechnology (Dallas, TX). 1,2-Distearoylsn-glycero-3-phosphoethanolamine-N-[FITC (polyethylene glycol)] (DSPE-PEG-FITC) was obtained from Ruixi Company (Xi’an, China). Tween 20 and lecithin were purchased from Solarbio Sciences and Technology (Beijing, China). Perfluorohexane (PFH, C_6_F_14_) was purchased from Macklin Biochemical (Shanghai, China). RPMI-1640, fetal bovine serum (FBS), 0.25% trypsin–EDTA, phosphate-buffered saline (PBS, pH 7.4), and antibiotics (100 U/mL penicillin and 100 mg/mL streptomycin) were obtained from Gibco Company (Brooklyn, NY). RNA mini Prep kit, mRNA reverse transcription systems, and RT-PCR master mix were obtained from Qiagen (Frederick, MD). Deionized or distilled water was used in all experiments. All other chemicals and reagents were of analytical grade.

### Cell lines and cell culture

The prostate cancer cell lines (22RV1 and PC-3) and bronchial epithelial cells (16HBE) were acquired from Cheeloo College of Medicine, Shandong University. In this study, the use of these cell lines was approved by the Research Ethics Committee of Qilu Hospital of Shandong University. Cells were cultured in complete medium (RPMI-1640 containing 10% FBS and 1% antibiotics), and maintained in incubator at 37 °C with 5% CO_2_ environment and 98% humidity.

### 3WJ-pRNA nanoparticles design, synthesis, and self-assembly

The four RNA segments (Table 1) were chemically synthesized (Sangon Biotech, Shanghai, China), strands 1–3 were 2′-F modified at uracil (U) and cytosine (C) nucleotides to make the RNA fragments resistant against RNase degradation. With the design on every strand, these RNA fragments possessed cholesterol for NDs anchoring (strand 1), A10-3.2 aptamers as a targeting ligand (strand 2), and siCAT-1 target CAT-1 as the therapeutic agent (strand 3 and strand 4). Cy3 as a fluorescence probe was added to the 3′ ends of strand 3.

**Table 1.** The RNA strands for constructing 3WJ-pRNA nanoparticles.

Strands	Modification	Sequence
Strand 1	aWJ-Cholesterol	5′-uuGccAuGuGuAuGuGGG-Cholesterol-3′
Strand 2	bWJ-A10-3.2	5′-cccAcAuAcuuuGuuGAuccGGGAGGAcGAuGcGGAucAGccA uGuuuAcGucAcuccu-3′
Strand 3	cWJ-siCAT-1	5′-GGAucAAucAuGGcAAuuGAcAuAuucGcAGuGAucAuAuu-cy3-3′
Strand 4	siCAT-1 antisense	5′-UAUGAUCACUGCGAAUAUGUCUU-3′

The core sequences of 3WJ-pRNA are shown by underline, lowercase letters indicate 2′-F modified nucleotides.

Assembly of 3WJ-pRNA nanoparticles (Chol/A10-3.2/siCAT-1/3WJ-pRNA) was accomplished by mixing the RNA fragments at equimolar amounts in 1× RNA annealing buffer (10 mM TRIS pH = 7.5, 50 mM NaCl, 1 mM EDTA) and annealing at 90 °C for 5 min followed by slow cooling to 4 °C more than 50 min on a PCR machine. 3WJ-pRNA nanoparticles were verified by 12% native-PAGE in running buffer (89 mM Tris, 200 mM boric acid, and 5 mM MgCl_2_). Gels were stained with ethidium bromide (EtBr), and then detected with Typhoon FLA 7000 (GE Healthcare, Chicago, IL). The morphology images were processed using the NanoScope Analysis software after imaging by a MultiMode AFM Nanoscope IV system (Bruker Instruments, Billerica, MA).

### Development and characterization of NDs

For the realization of echogenic property, PFH was incorporated into the NDs by the method of oil in water (O/W) emulsification. In short, a set amount of PFH (150 μL), lecithin (0.5 mg) and Tween 20 (6 μL) were homogenized in deionized water (2.5 mL) at 15,000 rpm for 2 min under the ice bath. O-CMC (5 mg) and DSPE-PEG (2.5 mg) were dissolved in deionized water (2.5 mL), and cautiously and dropwise added into the above emulsion, and followed by a homogenization for another 2 min. The upper solution was collected after centrifugation at 500 rpm for 3 min, and then filtered with a 0.45 μm filter (Millipore, Billerica, MA). Finally, the solution was stored at 4 °C or lyophilized.

To observe the morphology, size, and polydispersity of NDs, a bright-field and fluorescence-field were recorded at ×1000 magnifications using OLYMPUS BX41 microscope (Olympus Corporation, Tokyo, Japan), transmission electron microscopy (TEM) (Hitachi, Shiga, Japan) was also used to detect the morphology of NDs. Zeta potential and polydispersity index (PDI) were detected by a Zetasizer Nano ZS90 analyzer (Malvern Instruments Ltd., Worcestershire, UK).

### 3WJ-pRNA loading into NDs

NDs and 3WJ-pRNA nanoparticles were mixed, followed by anchoring reaction for 30 minutes at room temperature. After anchoring reaction, 3WJ-pRNA anchoring NDs (A10-3.2/siCAT-1/3WJ-NDs) were obtained using centrifugal flotation methodology. In short, after centrifugation at 500 rpm for 5 min, the lower layer comprised the NDs that were combined with the 3WJ-pRNA, while the upper suspension comprised the free 3WJ-pRNA. The amount of the free 3WJ-pRNA in the upper layer was measured and calculated with a nucleic acid protein detector (NanoDrop2000, Thermo Scientific, Wilmington, DE). Encapsulation efficiency was calculated by formulas: encapsulation efficiency=(total 3WJ-pRNA – free 3WJ-pRNA)/total 3WJ-pRNA. In order to establish an optimal concentration ratio, different concentrations of 3WJ-pRNA (0.1, 0.5, 1.0, 1.5, 2.0, and 3.0 μM) and NDs (1.0 mg/mL,100 μL) were mixed in equal volumes, the encapsulation efficiency were then calculated. The experiment was repeated three times.

### Gel retardation assay

The gel retardation assay was used to confirm the 3WJ-pRNA loading into NDs. Chol/A10-3.2/siCAT-1/3WJ-pRNA (1.5 μmol, 100 μL) and NDs (2 mg/mL, 100 μL) were mixed and incubated for 30 min at room temperature. Pre and after anchoring incubation, the samples were collected separately and electrophoresed through a 1% (w/v) agarose gel. Electrophoresis was performed using 1× TAE buffer at 80 V for 20 min. Then, the gels were stained with EtBr, and visualized using a UV transilluminator (Typhoon FLA 7000, GE Healthcare, Chicago, IL).

### Bio-safety assay

To evaluate the bio-safety of the materials (scramble siRNA, O-CMC, DSPE-PEG, lecithin, PFH) used for NDs preparation, 22RV1 cells were seeded and cultured in 96-well plates (6 × 10^3^ cells per well) for 24 h. Then, the mixed materials were respectively resuspended in 1640 medium at a series of different concentrations (0, 0.01, 0.05, 0.1, 0.2, 0.5, and 1.0 mg/mL) and added into each well replacing previous medium. 24 h or 48 h later, the medium containing the mixed materials was discarded and replaced with fresh 1640 medium (100 μL) containing CCK-8 solution (10 μL). After subsequently culture for 1.5 h, the absorbance at 450 nm was detected using an Infinite F200 multimode plate reader (Tecan, Männedorf, Switzerland). The antitumor roles of O-CMC (0, 0.1, 0.2, 0.5, 1.0, 2.0, 3.0, and 4.0 mg/mL) were measured using the similar method to the above, normal bronchial epithelial cells (16HBE) as controls. The experiments were repeated three times.

### Buffering capacity

The buffering capacity of NDs was evaluated over a pH range of 3–10 using an acid–base titration as described in a previous method (Garg et al., 2013) with minor modification. Briefly, a suspension of NDs (1 mg/mL, 10 mL) with shell of O-CMC/DSPE-PEG, O-CMC, and DSPE-PEG was prepared, respectively. The pH of the suspension was brought to 10 with 1.0 M sodium hydroxide (NaOH) at room temperature. 0.9% sodium chloride (NaCl) was used as negative control. Second, each solution was titrated by incrementally dropping 0.1 M HCl (50 μL each time) until pH of the suspension reached below 3, and the changes of pH were detected by pH meter (INESA Scientific Instrument PHB-4, Shanghai, China).

### siRNA transfection

siRNA for CAT-1 and scramble control were synthesized by RiboBio Company (Guangzhou, China). 22RV1 cells were seeded and cultured in six-well plates (2 × 10^5^ cells per well) for 24 h, and siRNAs were transfected into cells at a final concentration of 50 nM using Lipofectamine 2000. After the cells were transfected for 24 h, gene knockdown efficiency was evaluated using RT-PCR. The sequences were as follows:

- CAT-1 siRNA 1, 5′-CAGCUUACCUCUACAGCUAUG-3′;
- CAT-1 siRNA 2, 5′-GAGCAGCCUAACCUGGUAUAC-3′;
- CAT-1 siRNA 3, 5′-GACAUAUUCGCAGUGAUCAUA-3′;
- Scrambled siRNA, 5′-UUCUCCGAACGUGUCACGU-3′.

### Contrast-enhanced ultrasound imaging of NDs

CEUI of NDs was performed using an ultrasonic diagnostic apparatus with transducer frequency 7–10 MHz (LOGIQ E9; GE, Chicago, IL). Briefly, the NDs sample was resuspended with degassed deionized (DDI) water, and then injected into a plastic pipette (1 cm in diameter). The plastic pipette was fixed on the iron platform in 37 °C water bath. The parameter settings were as follows: center frequency of 9.0 MHz; mechanical index of 50%; dynamic range of 60 dB. At every different time point, a 10-s video was recorded and analyzed by the ‘TIC analysis’ function in the ultrasonic diagnostic apparatus. The CEUI intensity of NDs was normalized to that of the DDI water. The experiments were repeated three times.

### Cell migration assays

Cell migration assays were carried out in a transwell chamber. Briefly, cells were starved with serum free medium for 24 h and seeded in the upper chamber (2.5 × 10^4^ cells, 200 μL serum-free medium). The upper chamber was then placed into the bottom chamber containing 500 μL of culture medium. After incubation for 24 h, the cells on the upper surface of chambers were removed using cotton swabs. Cells on the bottom surface were fixed with polyoxymethylene and stained with 0.1% crystal violet for 20 minutes. The migratory ability was calculated through counting the cells on the bottom surface in five randomly fields under microscopy. The experiments were repeated three times.

### Cell targeting study of NDs on 22RV1 cells

To evaluate the targeting ability of A10-3.2/siCAT-1/3WJ-NDs, 22RV1 cells were seeded into specific cell climbing films (1 × 10^5^ cells per well) for fluorescence observation. After adhesion for 24 h, targeting A10-3.2/siCAT-1/3WJ-NDs and non-targeting siCAT-1/3WJ-NDs were dispersed in 1640 medium, then added into climbing films respectively and co-incubated for 1 h. All the cells were fixed with paraformaldehyde and dyed with DAPI. Finally, the climbing films were observed with inverted fluorescence microscope.

### Statistical analysis

All experimental data were statistically analyzed with SPSS 22.0 software (SPSS Inc., Chicago, IL). One-way ANOVA and Student’s *t*-test were performed to determine the statistical significance among groups. All data were expressed as mean ± standard deviation, *p* values of less than .05 suggested significant difference (*p< .05).

## Results

### Construction and characterization of Chol/A10-3.2/siCAT-1/3WJ-pRNA nanoparticles

**Figure 1.**
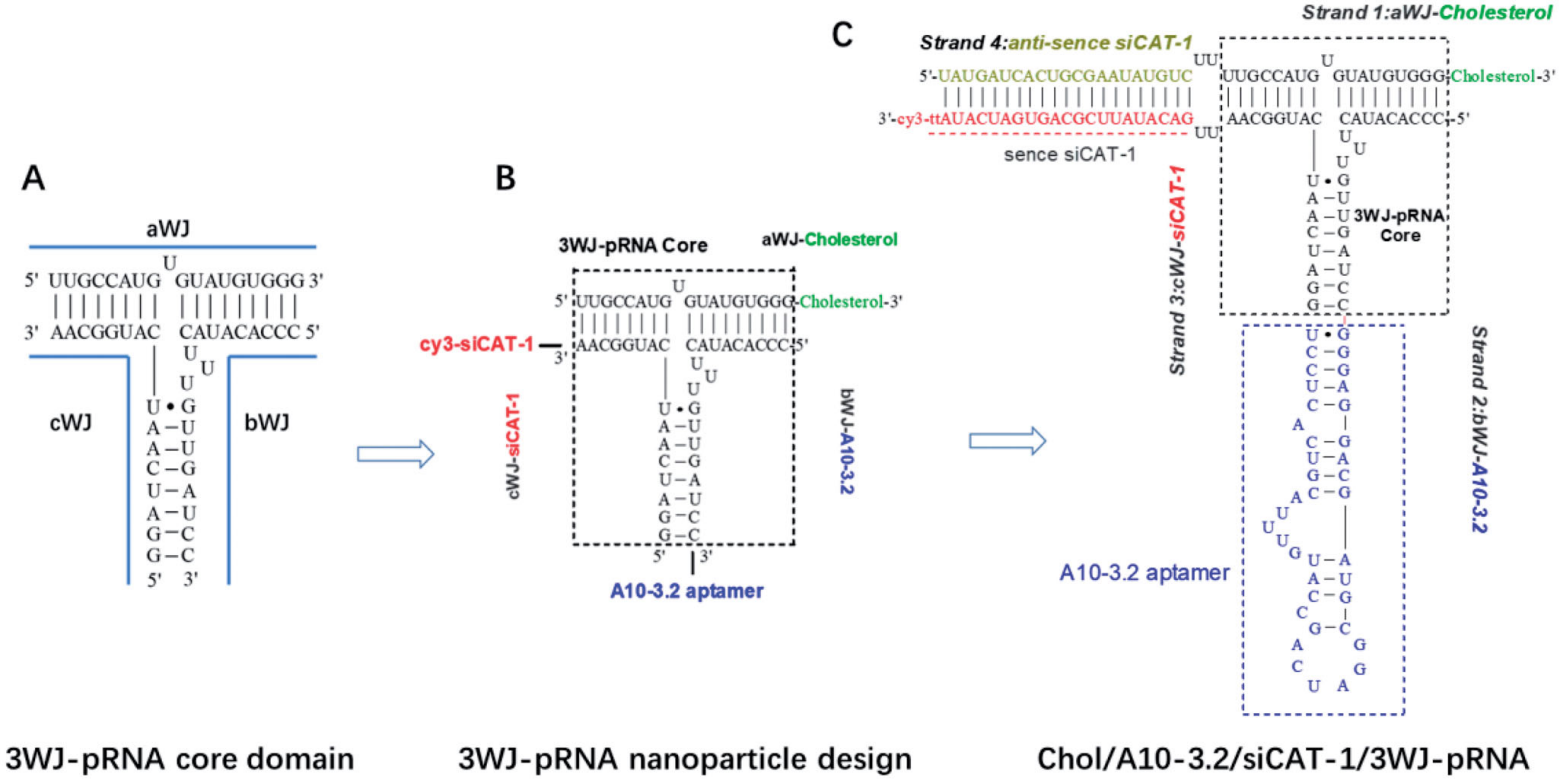
Design of Chol/A10-3.2/siCAT-1/3WJ-pRNA nanoparticles harboring cholesterol, A10-3.2 aptamer and siCAT-1 using 3WJ-pRNA as scaffold. (A) The sequence and secondary structure of 3WJ-pRNA core. aWJ, bWJ, and cWJ represent the three strands of the 3WJ-pRNA complex. (B) Design strategy of Chol/A10-3.2/siCAT-1/3WJ-pRNA nanoparticles harboring cholesterol for NDs-anchoring, A10-3.2 aptamer for PSMA-binding and siCAT-1 for CAT-1 silencing. (C) The detailed sequence and secondary structure of Chol/A10-3.2/siCAT-1/3WJ-pRNA, the 3WJ-pRNA core was boxed.

**Figure 2.**
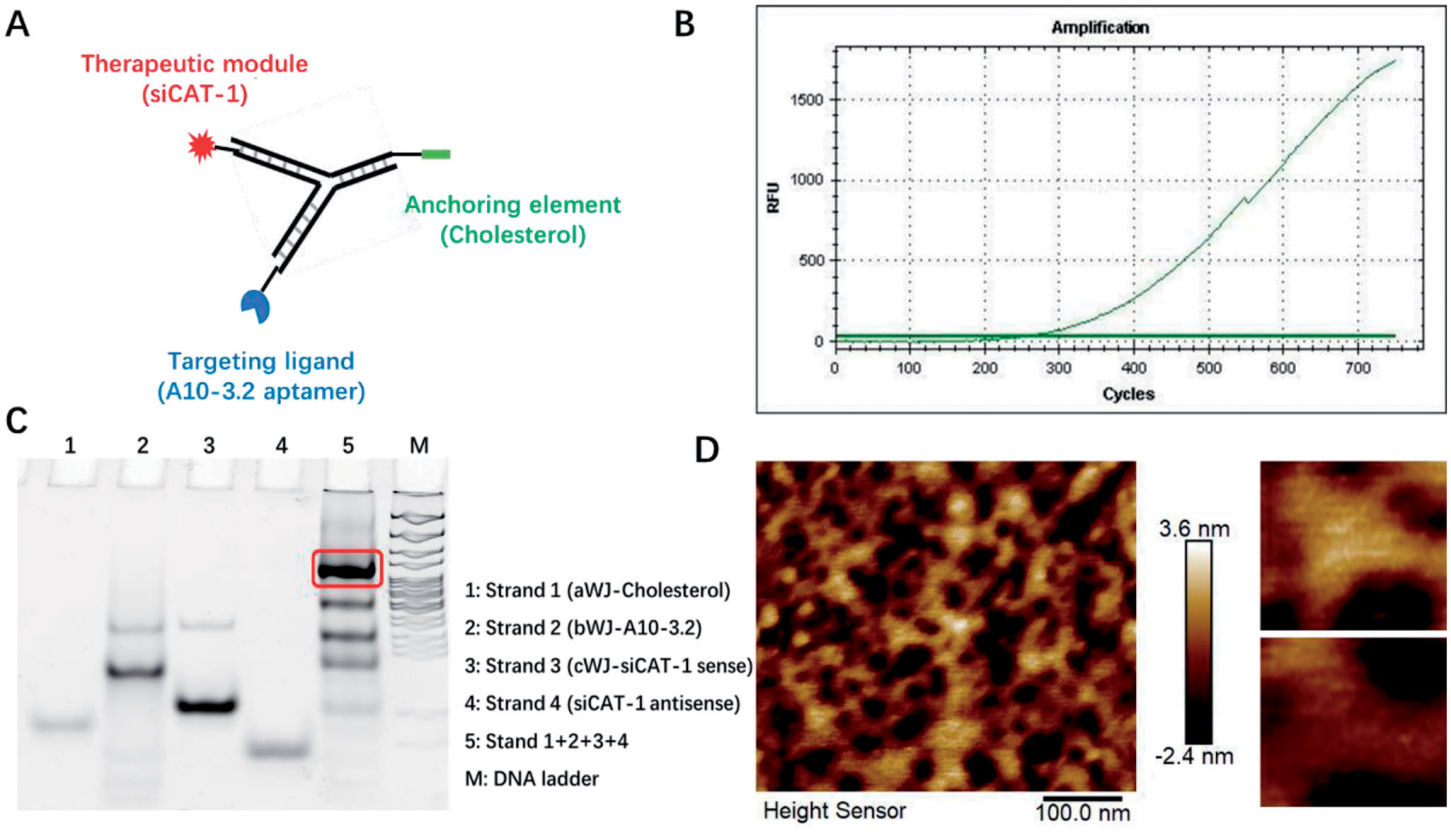
Physicochemical characterization of Chol/A10-3.2/siCAT-1/3WJ-pRNA nanoparticles. (A) Schematic showing Chol/A10-3.2/siCAT-1/3WJ-pRNA nanoparticles consisting of three modules bound at the 3WJ-pRNA core sequence (green, blue, and red). (B) Fluorescence intensity of SYBR green I for the assembly of the Chol/A10-3.2/siCAT-1/3WJ-pRNA. (C) Twelve percent native PAGE showing the assembly of the Chol/A10-3.2/siCAT-1/3WJ-pRNA. (D) AFM image showing triangular structure of self-assembled Chol/A10-3.2/siCAT-1/3WJ-pRNA.

In the search for an siRNA suitable for *CAT-1* mRNA knockdown, we tested three siRNA sequences and found that the CAT-1 siRNA-3 (hereinafter named as siCAT-1) could knockdown *CAT-1* expression with the highest efficiency. The 3WJ motif from 3WJ-pRNA nanoparticles can be used as a scaffold for the purpose of simultaneously harboring different modules such as tumor targeting ligand, therapeutic siRNA and imaging probe. In this study, we used 3WJ-pRNA as a scaffold and constructed trifunctional nanoparticles Chol/A10-3.2/siCAT-1/3WJ-pRNA, harboring PSMA-targeting module of RNA aptamer A10-3.2, therapeutic module of Cy3-labeled siCAT-1, anchoring module of cholesterol (Figures 1 and 2(A)). Upon mixing the corresponding RNA strands at equal molar ratio in annealing buffer, the 3WJ-pRNA nanoparticles assemble with high efficiency as indicated by fluorescence curve (Figure 2(B)). Native-PAGE gels showed high yield of the constructed 3WJ-pRNA nanoparticles, with some side product bands due to mismatch during self-assembly (Figure 2(C)), and the formation of three-way junction architecture was clearly demonstrated by the atomic force microscopy (AFM) images (Figure 2(D)).

### Development and characterization of A10-3.2/siCAT-1/3WJ-NDs

**Figure 3.**
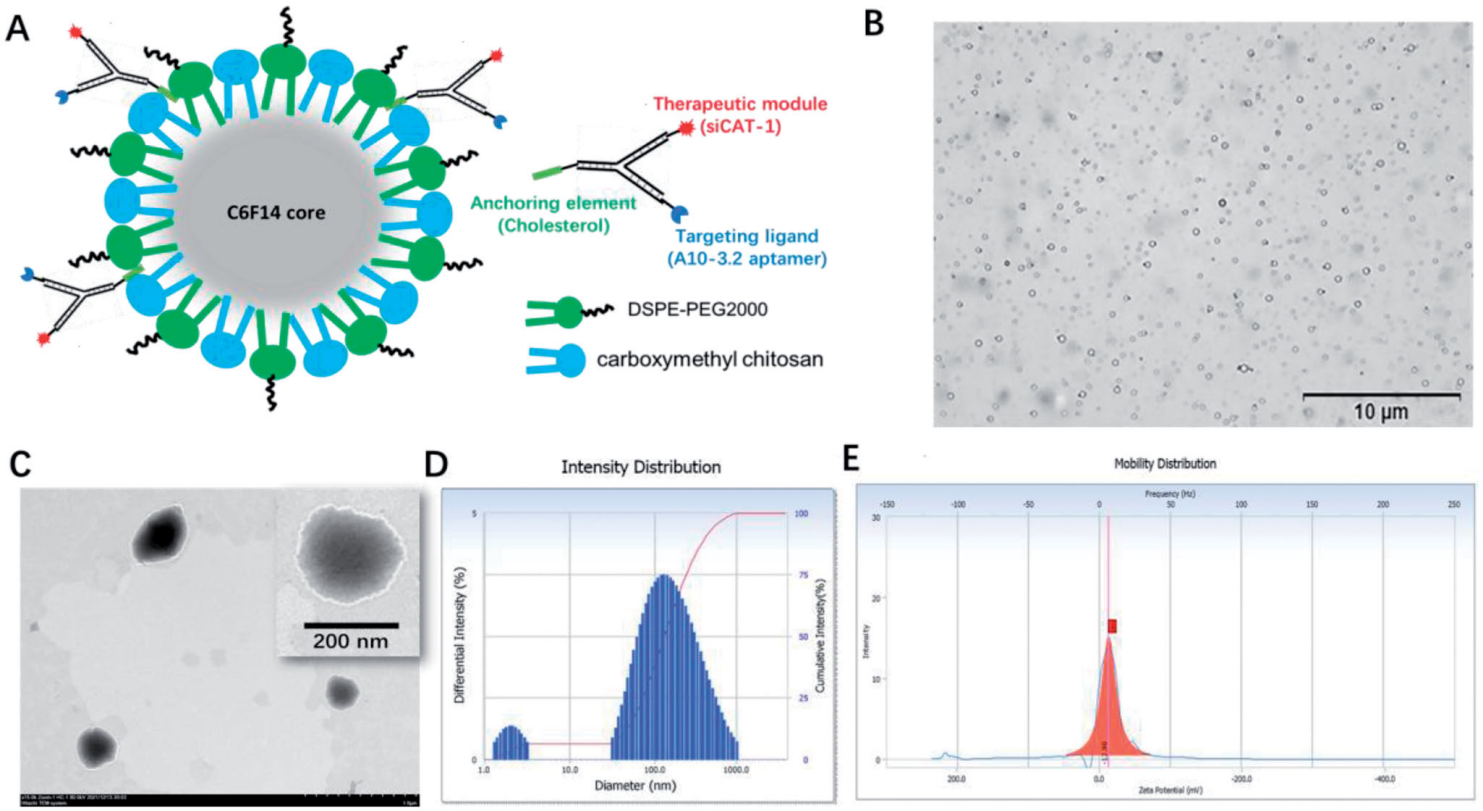
Characterization of A10-3.2/siCAT-1/3WJ-NDs. (A) Schematic illustration of A10-3.2/siCAT-1/3WJ-ND. (B) Morphology of NDs by inverted light microscope. (C) Morphology of NDs by transmission electron microscope (TEM). (D) Dynamic light scattering (DLS) data showing the size of A10-3.2/siCAT-1/3WJ-ND. (E) Zeta potential of A10-3.2/siCAT-1/3WJ-ND.

The US-responsive and negatively charged NDs were first developed with a method of homogenization/emulsion. As illustrated in Figure 3(A), the shell membrane of these NDs was made of O-CMC and DSPE-PEG, whereas PFH was used as the core. A10-3.2/siCAT-1/3WJ-NDs were then developed with these NDs and Chol/A10-3.2/siCAT-1/3WJ-pRNA through an anchoring strategy based on the cholesterol from the 3WJ-pRNA nanoparticles.

**Figure 4.**
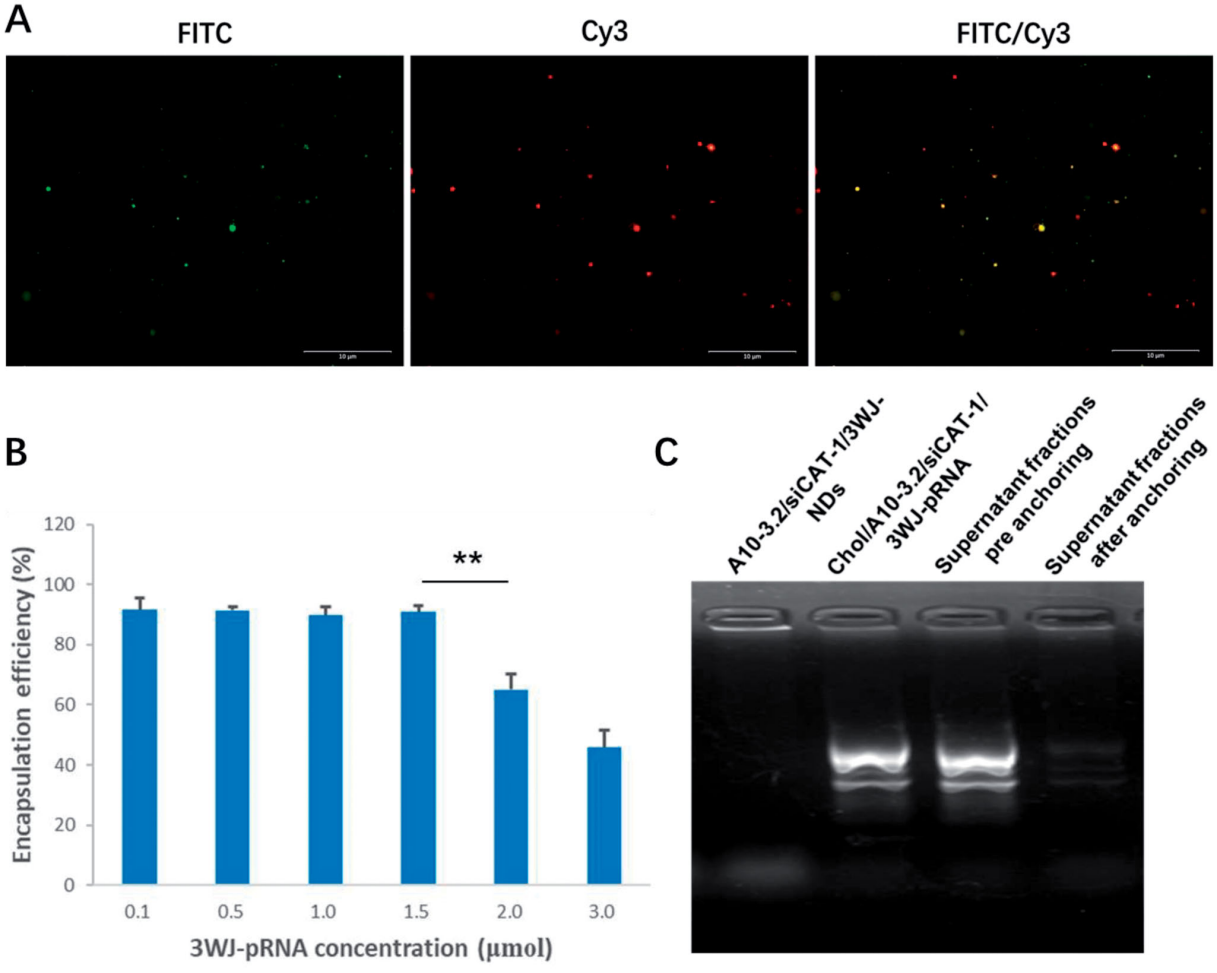
Loading 3WJ-pRNA into NDs. (A) Fluorescence characterization of A10-3.2/siCAT-1/3WJ-NDs, green fluorescence from FITC-labeled NDs, red fluorescence from Cy3-labeled Chol/A10-3.2/siCAT-1/3WJ-pRNA nanoparticles. (B) Encapsulation efficiency of Chol/A10-3.2/siCAT-1/3WJ-pRNA nanoparticles in A10-3.2/siCAT-1/3WJ-NDs. (C) Gel retardation assay to evaluate the loading efficiency of Chol/A10-3.2/siCAT-1/3WJ-pRNA. **p<.01.

Under light microscope and TEM, A10-3.2/siCAT-1/3WJ-NDs displayed round, spherical shapes, and core–shell structures (Figure 3(B,C)). According to dynamic light scattering (DLS) measurements (Figure 3(D,E)), the precise mean diameter of A10-3.2/siCAT-1/3WJ-NDs is about 198 nm (PDI: 0.3), and the zeta potential was −12.98 mV. Under an inverted fluorescence microscope, images of A10-3.2/siCAT-1/3WJ-NDs showed intact, discrete, and color spherical outlines, which was consistent with the FITC (green, FITC-labeled NDs) and Cy3 (red, Cy3-labeled Chol/A10-3.2/siCAT-1/3WJ-pRNA) (Figure 4(A)). From the merged FITC/Cy3 image (yellow), it can be seen that Chol/A10-3.2/siCAT-1/3WJ-pRNA nanoparticles (red) were successfully anchored onto NDs (green).

In order to establish an optimal 3WJ-pRNA concentration in preparing A10-3.2/siCAT-1/3WJ-NDs, the influences of initial Chol/A10-3.2/siCAT-1/3WJ-pRNA concentration on the encapsulation efficiency were performed. From the results of Figure 4(B), the encapsulation efficiency of 3WJ-pRNA decreased significantly at a concentration of 2.0 μmol, while no changes were noted when the concentration is below 1.5 μmol; therefore, a concentration of 1.5 μmol is optimal. Due to pore size restriction, Chol/A10-3.2/siCAT-1/3WJ-pRNA loaded in NDs was barely able to run into the gel (Figure 4(C), lane 1), while unloaded Chol/A10-3.2/siCAT-1/3WJ-pRNA can run into the gel under electrophoretic forces (Figure 4(C), lane 2). About 90% decrease of the gray value in the supernatant fractions was seen after anchoring incubation compared to ‘pre anchoring’ control groups (Figure 4(C), lanes 3 and 4), this result consistent with the encapsulation efficiency is shown in Figure 4(B).

### Contrast-enhanced ultrasound imaging ability of NDs

**Figure 5.**
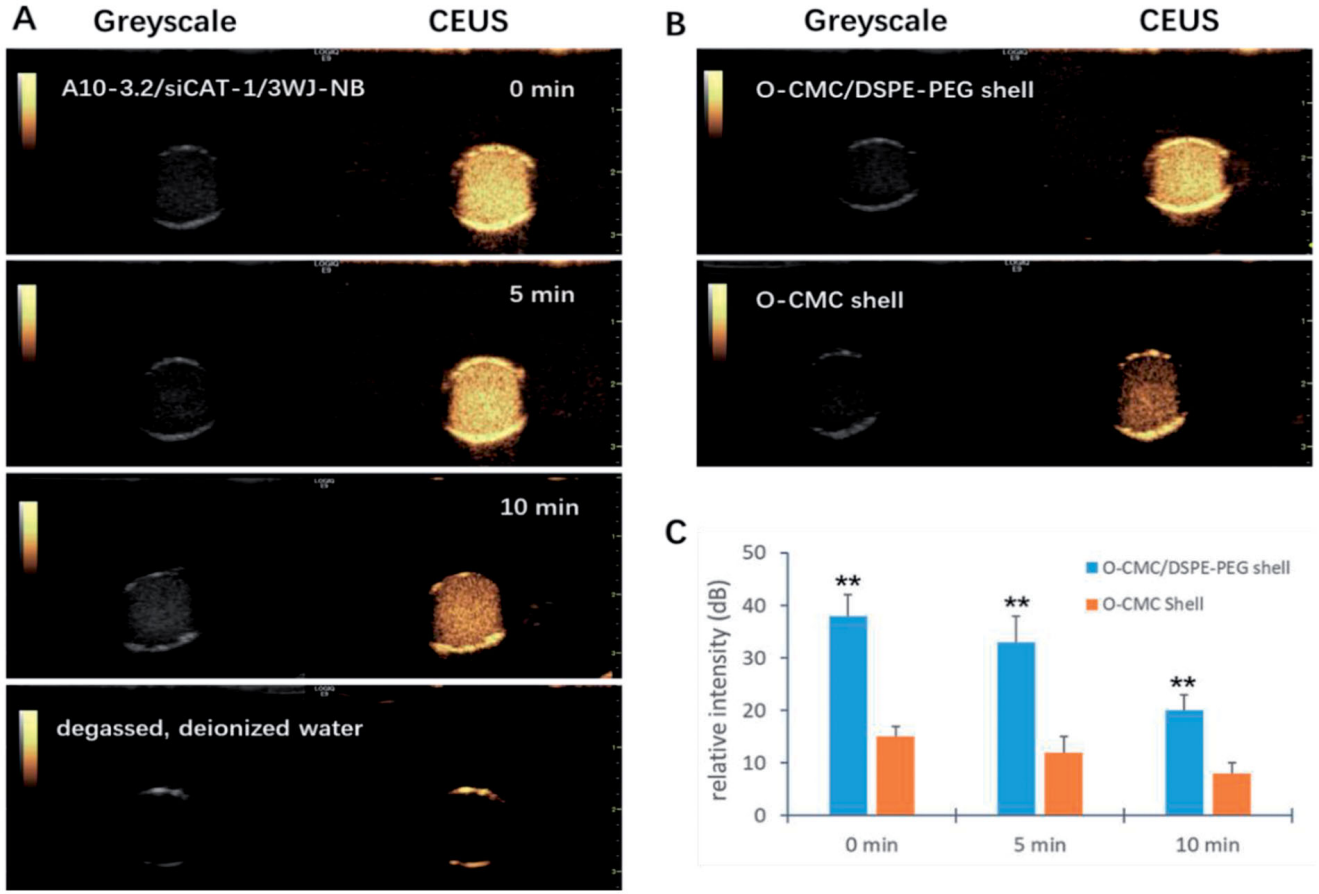
CEUI ability of NDs. (A) The CEUI ability of A10-3.2/siCAT-1/3WJ-NDs at the time points of 0 min, 5 min, and 10 min, DDI water used as negative control. (B) Comparison of the maximum intensities between O-CMC/DSPE-PEG (shell) NDs and O-CMC (shell) NDs. (C) The contrast intensity changes of NDs at different time point. **p<.01.

To be a potential US-responsive delivery system, CEUI ability should be one critical function for these novel NDs. Therefore, *in vitro* CEUI was carried out with an ultrasonic diagnostic apparatus (GE LOGEQ E9, Chicago, IL) at center frequency of 9.0 MHz and mechanical index of 0.5. The CEUI intensities of the NDs were continuously monitored no less than 10 min to analyze the imaging signal changes. As shown in Figure 5(A), these NDs showed excellent CEUI ability with DDI water as the negative control. After 5 min, the CEUI intensities were a little dimmer compared with before (*p*>.05). At the time point of 10 min, a significant reduction of CEUI intensity was seen compared with that of 0 min and 5 min (*p*<.05). The CEUI intensities of these NDs at the time point of 0 min, 5 min, and 10 min were 38.24 ± 0.32 dB, 33.95 ± 0.27 dB, and 20.39 ± 0.25 dB, respectively. O-CMC/DSPE-PEG NDs exhibited higher CEUI intensity than O-CMC NDs whether at 0 min, 5 min, and 10 min (Figure 5(B,C), *p*<.01). These results suggest that O-CMC/DSPE-PEG NDs could keep stable during long time for US imaging.

### Buffering capacity of NDs

**Figure 6.**
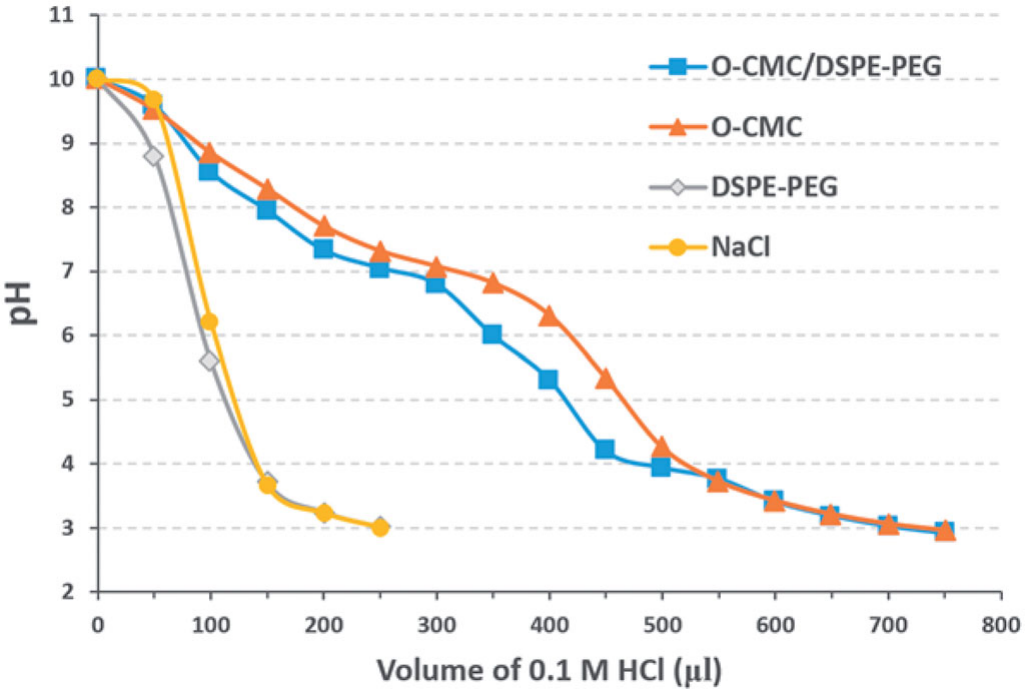
Buffering capacity of NDs. 0.1 M HCl was dropped into the mediums from O-CMC/DSPE-PEG, O-CMC, DSPE-PEG, and pH values were recorded from 10 to 3. Meanwhile, NaCl solution was used as the negative control.

An adequate buffering capability of NDs is required for its endosomal escape and effective siRNA delivery. As shown in Figure 6, the NDs with shell of O-CMC/DSPE-PEG or O-CMC displayed a slower downtrend and gentler slope in the titration curve than the NDs with shell of DSPE-PEG and the negative control of NaCl over the pH range 7.4–5.0, indicating that the NDs with shell of O-CMC/DSPE-PEG exhibited a good buffering capacity.

### *In vitro* bio-safety of NDs

**Figure 7.**
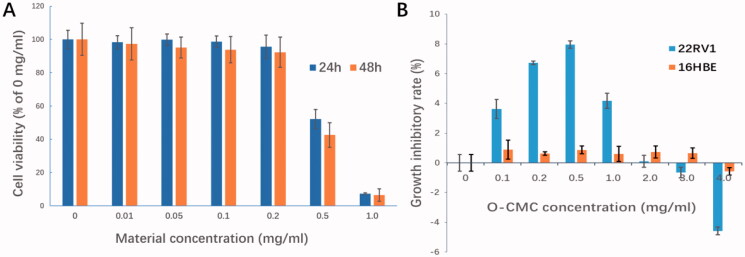
*In vitro* bio-safety of NDs. (A) Effect of materials used for NDs construction on 22RV1 cell viability was evaluated by CCK-8 cytotoxicity assay. (B) Growth inhibitory effect of O-CMC on 22RV1 cells was assessed by CCK-8 assay, non-tumor 16HBE cells used as negative control.

The bio-safety was performed by incubating the 22RV1 cells with the materials used for NDs preparation at series concentrations (0, 0.01, 0.05, 0.1, 0.2, 0.5, and 1.0 mg/mL) using CCK-8 assay. The cytotoxicity appeared obviously when the materials concentration was up to 0.5 mg/mL (*p*< .01, compared with other concentrations, Figure 7(A)). At used concentration for cell experiments in this study (<0.2 mg/mL), the cell survival rate was all above 95%, which indicates that these NDs have high bio-safety.

Unlike lipids, which is known to be less cytotoxicity, chitosan is generally accepted as a potential anti-tumor agent. However, it is still not clear whether the O-CMC (derivative of chitosan) has the similar capacity. In this study, the anti-tumor role of O-CMC was also analyzed. Interestingly, with the exposure concentration increasing, the anti-growth activities of O-CMC were induced initially and then decreased, even showed the activity of promoting tumor when the concentration was over 3.0 mg/mL (Figure 7(B)). In order to determine whether O-CMC has the similar anti-growth capacity of non-tumor cells, normal human bronchial epithelial cells (16HBE) were tested simultaneously, the results showed that O-CMC has a little anti-growth effect on 16HBE compared with 22RV1 cells (Figure 7(B), *p*<.01). These results suggest that O-CMC has a certain anti-tumor activity.

### Targeting of A10-3.2/siCAT-1/3WJ-NDs to 22RV1 cells

**Figure 8.**
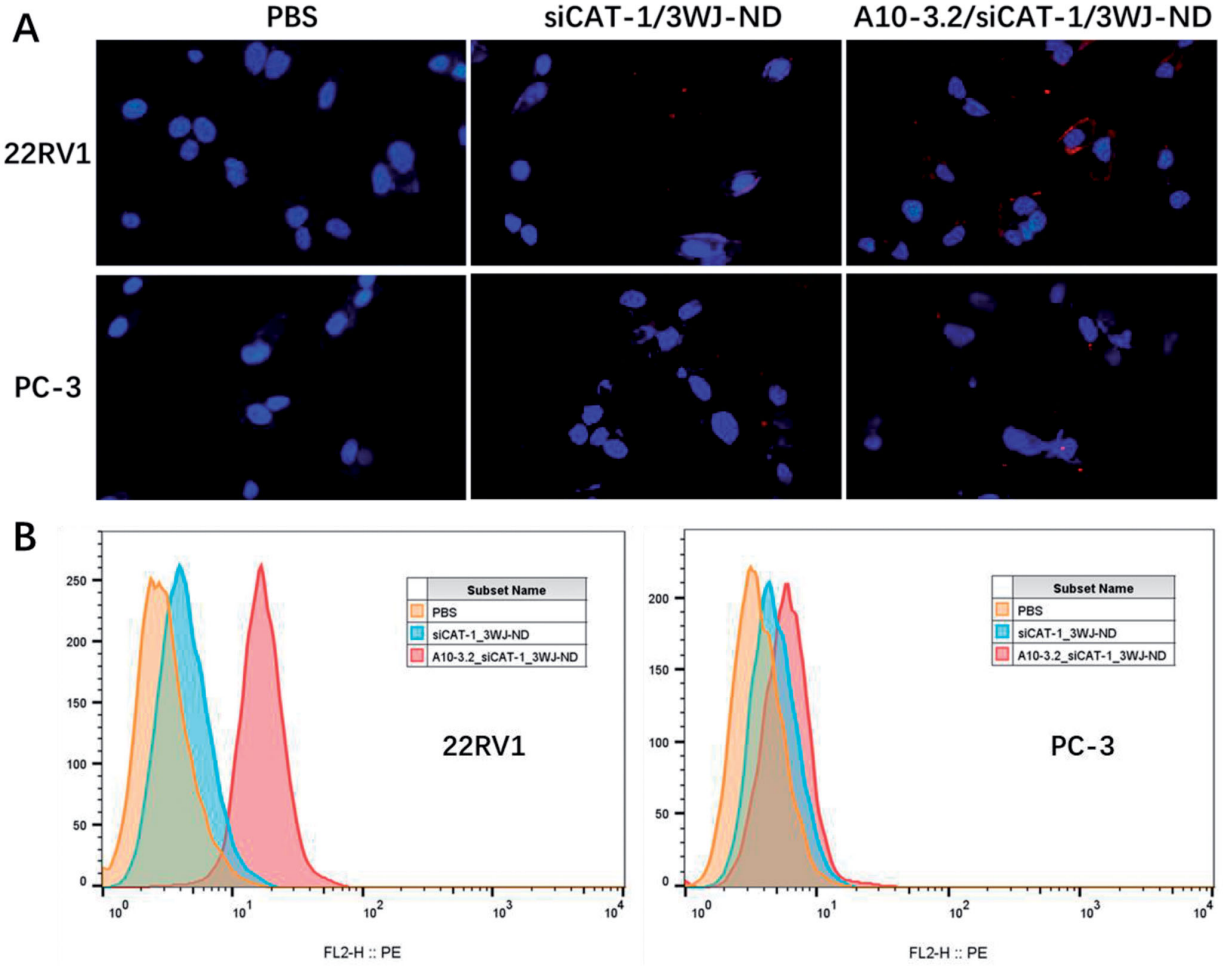
Targeting binding of NDs to 22RV1 cells. (A) Targeting binding of non-targeted siCAT-1/3WJ-NDs and targeted A10-3.2/siCAT-1/3WJ-NDs to PSMA(+) 22RV1 cells by fluorescence microscope, PSMA(–) PC-3 cells used as control. (B) Corresponding flow cytometry analysis of the above groups.

Specific cancer cell targeting is an important prerequisite for applying these NDs to tumor diagnosis and treatment. In order to test the targeting ability of A10-3.2/siCAT-1/3WJ-NDs, siCAT-1 was labeled with Cy3 fluorophore to allow for tracking of these NDs. The targeting capabilities were examined using PSMA-positive 22RV1 cells and control PSMA-negative PC-3 cells, as well as siCAT-1/3WJ-NDs (without any targeting module) as a negative control. Following the NDs co-incubation, cells were detected by fluorescence microscope and flow cytometry for confirming the targeting binding of NDs. We found that A10-3.2/siCAT-1/3WJ-NDs can efficiently bind with 22RV1 cells but not PC-3 cells, while siCAT-1/3WJ-NDs were little to be found either in 22RV1 cells or PC-3 cells (Figure 8(A)). Flow cytometry data further determined that A10-3.2/siCAT-1/3WJ-NDs were effectively taken up by 22RV1 cells rather than PC-3 cells (92.3% vs. 7.52%), and siCAT-1/3WJ-NDs cannot noticeably target these two cell lines (Figure 8(B)). Together, these results demonstrated that A10-3.2/siCAT-1/3WJ-NDs could effectively target to PSMA-positive prostate cancer cells.

### Delivery of siCAT-1 to 22RV1 cells

**Figure 9.**
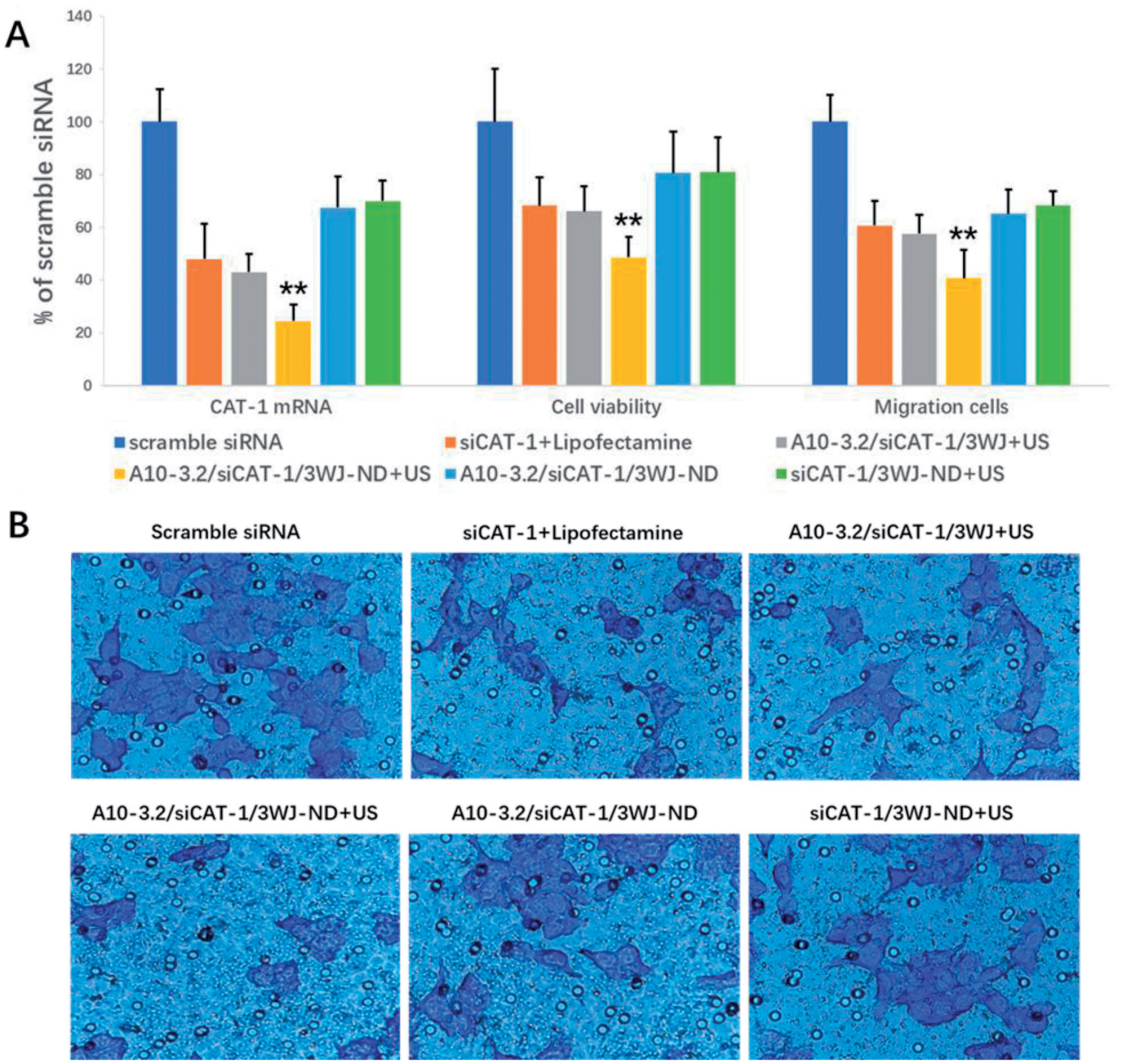
Targeted delivery of siCAT-1 to 22RV1 cells by A10-3.2/siCAT-1/3WJ-NDs and ultrasound irradiation. (A) After various treatments, the expression of *CAT-1* mRNA was examined using quantitative RT-PCR, the cell viability was assessed using CCK-8, and the cell migration was detected by transwell assay. (B) Representative light photomicrographs of migrated 22RV1 cells in various groups. **p<.01.

Small interfering RNA targeting *CAT-1* mRNA (siCAT-1) was placed onto the branch strand of 3WJ-pRNA for delivery into 22RV1 cells. The abilities of A10-3.2/siCAT-1/3WJ-NDs to delivery of siCAT-1 and silence the expression of *CAT-1* mRNA were examined using quantitative RT-PCR, keeping the siRNA concentration of various groups in the same level (50 nM). As shown in Figure 9(A), data show significant decrease in *CAT-1* expression in the A10-3.2/siCAT-1/3WJ-NDs + US group (24.37% of scramble siRNA), which had significant difference comparing with that in siCAT-1/3WJ-NDs + US group (no targeting, 69.89% of scramble siRNA), A10-3.2/siCAT-1/3WJ-NDs group (no US, 67.36% of scramble siRNA), A10-3.2/siCAT-1/3WJ + US group (no NDs, 42.93% of scramble siRNA), and siCAT-1 + Lipofectamine group (no NDs and US, 47.96% of scramble siRNA). These results show the siCAT-1 sequences loaded with A10-3.2/siCAT-1/3WJ-NDs were effectively delivered to 22RV1 cells with the help of A10-3.2 aptamer, ultrasonic NDs, and US irradiation. Due to the down-regulation of CAT-1 expression, the inhibitory effects on the growth of 22RV1 cells in various groups were in the order of A10-3.2/siCAT-1/3WJ-NDs + US > A10-3.2/siCAT-1/3WJ + US > siCAT-1 + Lipofectamin > A10-3.2/siCAT-1/3WJ-NDs > siCAT-1/3WJ-NDs + US > scramble siRNA, correspondingly. The inhibitory effects on the migration of 22RV1 cells in various groups were in the same order, as shown in Figure 9(B).

## Discussion

The successful application of siRNA for tumor treatment is limited by low transfection efficiency, susceptibility to degradation, and cationic toxicity of positive-charge nanocarriers (Charbe et al., 2020). Hence, development of an appropriate delivery system to improve the treatment efficiency of siRNA is urgently needed. In this work, we for the first time employed an innovative negatively charged and US-responsive NDs through 3WJ-pRNA nanotechnology to improve delivery efficiency of siRNA to tumor cells.

Cholesterol was conjugated into the aWJ branch for promoting the anchorage of 3WJ-pRNA nanoparticles onto NDs membrane. Cholesterol can spontaneously insert into the membrane of extracellular vesicles through its hydrophobic moiety, and cholesterol-modified siRNAs are becoming a simple, easy, scalable, and generally used method to load with siRNA cargo (Haraszti et al., 2018; Li et al., 2018). Under this guidance of theoretical basis, there is a reasonable prospect that cholesterol-modified 3WJ-pRNA nanoparticles can also anchor onto the NDs with a hydrophobic core similar to the extracellular vesicles, and the results of this research confirmed our predictions. Membrane-anchoring cholesterol was placed at the arrow-tail rather than arrow-head of aWJ brand in this study. It is because the arrow-tail configuration could form a 60° angle by two arms, which can act as a hook to prevent the 3WJ-pRNA nanoparticles from trafficking inside NDs, ensuring outer surface display of RNA aptamer for tumor receptor binding (Li et al., 2018; Pi et al., 2018).

In order to achieving specific targeting of NDs to tumor cells, one PSMA targeting ligand, RNA aptamer (A10-3.2) was conjugated to the bWJ branch of 3WJ-pRNA for displaying on the NDs surface. The expression of PSMA is associated with more aggressive diseases in prostate cancer (Nauseef et al., 2021). Many PSMA ligands have been developed, such as aptamers, engineered antibodies, and monoclonal antibodies. Among these targeted ligands, aptamers composed of nucleotides or deoxynucleotide have advantages compared with other ligands such as strong specificity, low-immunogenicity, and non-toxicity (Wu et al., 2017; Nauseef et al., 2021). As the above advantages, aptamers have been extensively applied in molecular imaging and targeted therapies. Herein, RNA aptamer A10-3.2 was conjugated to the 3WJ-pRNA nanoparticles, and then displayed on outer surface of NDs through cholesterol-mediated anchoring.

As a therapeutic module, siCAT-1 against CAT-1, was annealed to the cWJ branch of 3WJ-pRNA for tumor therapy. CAT-1, belongs to CAT family, which plays a major role in transporting l-arginine for the biosynthesis of nitric oxide and polyamine in mammalian cells, that are found to promote tumor cell growth and metastasis in types of tumors (Abdelmagid et al., 2011; Dai et al., 2017). Increasing studies indicates that targeting CAT-1 could be considered as a novel strategy for tumor-targeted therapy (Zhang et al., 2020). Therefore, CAT-1 was selected as a gene therapeutic target in this study. Cy3, a kind of anthocyanin dye, was attached to the siCAT-1 sequence, for detecting biological behavior of the NDs due to its red fluorescence under excitation.

With the above designs, 3WJ-pRNA nanoparticles are able to accommodate three modules at the same time for distinct purposes, including anchorage module (cholesterol), tumor targeting module (A10-3.2 aptamer) and gene therapeutic module (siCAT-1). Chol/A10-3.2/siCAT-1/3WJ-pRNA nanoparticles were constructed through methods of stepwise cooling as mentioned in earlier research (Zhang et al., 2017; Guo et al., 2018; Yoo et al., 2021). In order to confer RNase resistance, 2′-fluoro (2′-F) modifications at uracil and cytosine nucleotides were applied to the RNA sequence for 3WJ-pRNA construction. Successful construction of 3WJ-pRNA nanoparticles was confirmed by SYBR green I fluorescence curve, native-PAGE and AFM, as shown in Figure 2. Then, 3WJ-pRNA anchoring-NDs were then developed by simply co-incubation with cholesterol-modified 3WJ-pRNA nanoparticles and O-CMC/DSPE-PEG NDs. After decorating O-CMC/DSPE-PEG NDs with Chol/A10-3.2/siCAT-1/3WJ-pRNA nanoparticles, there was not an obvious change in size, as measured by DLS with 198 nm. The encapsulation efficiency for 3WJ-pRNA nanoparticles was approximately 90% as measured by free nucleic acid amount in the supernatant fractions.

In our previous articles, we constructed several kinds of US-responsive NDs using O-CMC (Meng et al., 2019; Shang et al., 2020), or DSPE-PEG (Duan et al., 2017; Guo et al., 2019; Sun et al., 2020) as shell membrane. Unlike before, we constructed the NDs using O-CMC/DSPE-PEG mixed materials as shell membrane instead of O-CMC alone or DSPE-PEG alone in this study. There have not been any related studies about O-CMC/DSPE-PEG mixed materials as the shell membrane of US-responsive NDs, so we analyzed some features such as CEUI and buffering capacity. The CEUI ability of these NDs is due to the core of PFH, despite the relatively high boiling temperature (58–60 °C), NDs with PFH core can be acoustically vaporized into microbubbles upon the US irradiation, and many literatures also have prepared PFH NDs for enhanced US imaging (Baghbani et al., 2017a, 2017b; Yu et al., 2018; Chang et al., 2019; Gao et al., 2021). The predominant principle for acoustic droplet vaporization (ADV) of PFH is not fully understood, possibly due to the mechanical role of US (Baghbani et al., 2017a, 2017b), and the precise principle should be further studied in the future. The results from Figure 5 showed that O-CMC/DSPE-PEG NDs exhibited higher CEUI than that of O-CMC NDs, and also can keep stable for long time at least 10 min. We hypothesize that DSPE-PEG may introduce elastic and compliant in the shell membrane, while O-CMC alone forms a shell that provides stiffness, limits strain during expansion and compression. Estimation of the buffering capacity was essential to determine whether NDs could induce endosomal escape through proton sponge effect and release the siRNAs into the cytoplasm (Huang et al., 2020). Here, we also determined the buffering capacity of these NDs, as shown in Figure 6, O-CMC/DSPE-PEG NDs were able to neutralize more HCl and exhibited greater buffering capacity, and O-CMC NDs had a similar buffering capacity, while DSPE-PEG NDs had no buffering capacity, which indicated that the buffering capacity of O-CMC/DSPE-PEG NDs is entirely because of the presence of amphoteric electrolyte (O-CMC). These results here provide a good choice to construct NDs, not only for CEUI, but also for siRNA delivery.

Owing to the advantageous characteristics like anti-tumor activity, non-toxicity, biodegradability and biocompatibility, chitosan has been recommended by US Food and Drug Administration (FDA) for wide application in targeted drug/gene delivery (Wang et al., 2020). Carboxymethyl chitosan is one derivative of chitosan, exhibiting numerous outstanding biological properties beside chitosan, such as water-soluble and pH-responsiveness (Shariatinia, 2018; Meng et al., 2019). Chitosan and its derivatives have been found to exhibit antitumor activity in many cancer models (Chi et al., 2019; Dong et al., 2019), so here we analyzed the tumor inhibition effects of O-CMC in prostate cancer 22RV1 cells. Consistent with previous investigations, the similar antitumor role was also obtained in 22RV1 cells, as Figure 7(B) shows. Although this anti-tumor activity is not enough strong, as a shell membrane material, this characteristic undoubtedly makes O-CMC as a potential and important choice for nanocarrier.

The targeting, delivery, and therapeutic efficiency of the A10-3.2/siCAT-1/3WJ-NDs were examined in PSMA-positive 22RV1 cells. PSMA aptamer-displaying A10-3.2/siCAT-1/3WJ-NDs showed enhanced binding to PSMA(+) 22RV1 cells compared to siCAT-1/3WJ-NDs without A10-3.2 aptamer by fluorescence microscope and flow cytometry analysis (Figure 8). Besides, studies performed in PC-3 cells showed no significantly targeting in A10-3.2/siCAT-1/3WJ-NDs group, suggesting these NDs were able to selectively targeting tumor cells through PSMA binding, indicating a low toxicity to PSMA(–) normal tissue. US irradiation is used frequently as a theranostic tool because of its convenience and non-invasiveness, US irradiation combination with US-responsive NDs can enhance the delivery of drug/nucleic acids via the sonoporation mechanism induced by US-mediated destruction and cavitation of NDs (Yang et al., 2020; Deprez et al., 2021). This is the advantage for US-responsive NDs compared with the other nanocarriers. In this study, under US irradiation, A10-3.2/siCAT-1/3WJ-NDs were able to knockdown the CAT-1 expression more obviously than that without US irradiation, and consequently suppressing 22RV1 cell proliferation and migration.

Since cationic toxicity remains the bottleneck for NDs-mediated siRNA delivery through electrostatic interactions; therefore, in the current study, we constructed negatively charged and US-responsive A10-3.2/siCAT-1/3WJ-NDs for siRNA-targeted delivery through 3WJ-pRNA nanotechnology. Through a series of experiments, it can be seen that these novel NDs were able to delivery siRNA to PSMA(+) prostate 22RV1 cells, and meanwhile exhibited good CEUI and high-biosafety. Despite lacking *in vivo* experiments, the methods of 3WJ-pRNA modification in this study will no doubt provide a novel strategy for negatively charged and US-responsive NDs-mediated siRNA delivery. Future studies will be needed to determine this strategy in other cancer cell models, or other RNA molecule delivery.

## Conclusions

Here, we report for the first time the development of negatively charged and US-responsive NDs through 3WJ-pRNA nanotechnology for specific delivery of siRNA to tumor cells. Cholesterol was conjugated into the aWJ branch to promote the anchorage of the 3WJ-pRNA nanoparticles onto the NDs outer interface. Targeting was achieved through PSMA-targeted A10-3.2 RNA aptamer, which conjugated into the bWJ branch. siCAT-1 as well as Cy3 fluorescent tags were conjugated into the cWJ branch for silencing CAT-1 expression and monitoring the targeting efficiency to 22RV1 cells. *In vitro* experiments showed that these reprogrammed A10-3.2/si-CAT-1/3WJ-NDs displayed good CEUI, enhanced tumor cell specific targeting, and increased intracellular delivery of siCAT-1 to suppress tumor growth. Through these studies, we have proven that the 3WJ-pRNA nanotechnology is an efficient modification strategy for negatively charged and US-responsive NDs-mediated siRNA delivery.

## Data Availability

The data that support the findings of this study are available from the corresponding author upon reasonable request.
